# Clinical characterization and therapeutic targets of vitamin A in patients with hepatocholangiocarcinoma and coronavirus disease

**DOI:** 10.18632/aging.203220

**Published:** 2021-06-27

**Authors:** Xiao Liang, Rui Zhou, Yu Li, Lu Yang, Min Su, Keng Po Lai

**Affiliations:** 1 Laboratory of Environmental Pollution and Integrative Omics, Guilin Medical University, Guilin, Guangxi, China; 2 Department of Hepatobiliary Surgery, Guigang City People's Hospital, The Eighth Affiliated Hospital of Guangxi Medical University, Guigang, Guangxi, China; 3 Guangxi Key Laboratory of Tumor Immunology and Microenvironmental Regulation, Guilin Medical University, Guilin, Guangxi, China

**Keywords:** hepatocholangiocarcinoma, coronavirus disease, COVID-19, vitamin A, vitamin A, network pharmacology

## Abstract

Recent reports indicate that patients with hepatocholangiocarcinoma (CHOL) have a higher morbidity and mortality rate for coronavirus disease (COVID-19). Anti-CHOL/COVID-19 medicines are inexistent. Vitamin A (VA) refers to a potent nutrient with anti-cytotoxic and anti-inflammatory actions. Therefore, this study aimed to determine the potential functions and molecular mechanisms of VA as a potential treatment for patients with both CHOL and COVID-19 (CHOL/COVID-19). The transcriptome data of CHOL patients were obtained from the Cancer Genome Analysis database. Furthermore, the network pharmacology approach and bioinformatics analysis were used to identify and reveal the molecular functions, therapeutic biotargets, and signaling of VA against CHOL/COVID-19. First, clinical findings identified the medical characteristics of CHOL patients with COVID-19, such as susceptibility gene, prognosis, recurrence, and survival rate. Anti-viral and anti-inflammatory pathways, and immunopotentiation were found as potential targets of VA against CHOL/COVID-19. These findings illustrated that VA may contribute to the clinical management of CHOL/COVID-19 achieved by induction of cell repair, suppression of oxidative stress and inflammatory reaction, and amelioration of immunity. Nine vital therapeutic targets (*BRD2, NOS2, GPT, MAPK1, CXCR3, ICAM1, CDK4, CAT,* and *TMPRSS13)* of VA against CHOL/COVID-19 were identified. For the first time, the potential pharmacological biotargets, function, and mechanism of action of VA in CHOL/COVID-19 were elucidated.

## INTRODUCTION

Coronavirus disease (COVID-19) is a worldwide spread disease, caused by severe acute respiratory syndrome coronavirus 2 (SARS-CoV-2) [1]. Increasing epidemiological data shows that the prevalence, mortality rate, and spread of COVID-19 is rising rapidly worldwide, especially in the United States [2]. There is currently no effective treatment for COVID-19 [3]. Despite the ongoing studies for the development of a COVID-19 vaccine by researchers, the clinical effectiveness of vaccine remains undetermined [4]. Accordingly, there is an urgent need for further research to develop effective bioactive ingredients to treat COVID-19. On the other hand, increasing evidence indicates that cancer patients may be high risk in SARS-CoV-2 infection, with a high death rate [5]. Hepatocholangiocarcinoma (CHOL) is a rare type of hepatic carcinoma characterized by its high invasion and metastasis potential [6]. According to the cancer statistics of China, liver cancer, including CHOL, is the leading cause of cancer-related deaths [7]. Hospitals are high-risk places for SARS-CoV-2 infection and transmission in early outbreaks due to the hard-to-diagnose symptoms of this new virus [8]. Accordingly, the CHOL patients in the hospital may have a higher risk of exposure to SARS-CoV-2. Therefore, it can be difficult to treat patients with both CHOL and COVID-19 (CHOL/COVID-19), and the fatality rate is high given the absence of an effective treatment [9–11]. Therefore, there is a need to develop a specific treatment targeting CHOL/COVID-19 patients.

Vitamin A (VA), a functional nutrient, is necessary for normal vision and has anti-inflammatory properties [12]. VA facilitates growth and reproduction, maintains bones and epithelial tissue, and aids in mucosal epithelium secretion [13]. Further, VA supplementation can prevent precancerous lesions [14]. In VA deficiency, the epithelial cells in the respiratory tract are keratinized, resulting in reduced immunity and an increased risk of infection [15]. VA regulates different gene targets through nuclear receptors, leading to improve immune system and induce the production of cytokines by immune cells [16, 17]. VA has antiviral and antitumor properties, as it is extremely important in maintaining a sufficient level of natural killer cells in circulating blood [18]. *In vitro* studies show that a high dose of VA may have anti-tumor effects in human cancer cell lines [19]. Clinical findings indicate that high-dose intake of VA may reduce the risk of liver cancer in the Chinese population [20]. However, the association between VA and CHOL remains unknown. In addition, the therapeutic action and mechanism of VA in CHOL/COVID-19 have still not been reported. As an attractive strategy, network pharmacology is an effectual approach for uncovering the putative, vital target, function, and pathway of bioactive ingredients against clinical disorders [21, 22]. Our previous bioinformatics findings revealed all vital targets, pharmacological functions, and molecular mechanisms of some bioactive compounds in complex diseases, including hepatic carcinoma, sepsis, and pneumonia [23–25]. Therefore, in this report, using the network pharmacology approach, we aimed to identify and characterize the mechanism underlying anti-CHOL/COVID-19 pharmacological activity of VA. The finding of this study would provide an alternative approach to use vitamin A as a supplement to boost up the efficiency of the existing vaccines for CHOL/COVID-19 treatment.

## MATERIALS AND METHODS

### Collection of CHOL/COVID-19-related genes

In order to determine the CHOL/COVID-19-associated genes, we obtained the transcriptome dataset of CHOL patients from the Cancer Genome Atlas (TCGA) database (https://portal.gdc.cancer.gov/) on July 25, 2020 as previous described [23]. The differentially expressed genes in CHOL patients were identified using the "limma" package of R language Bioconductor (with a false discovery rate <0.05 and |log^fold change (FC)^|> 1). Then, COVID-19-associated genes were obtained from different databases including the Genecard database, Online Mendelian Inheritance in Man (OMIM) database, and National Center for Biotechnology Information (NCBI) gene function module. Then, the CHOL- and COVID-19-associated genes were compared and overlapped as previous descried [26].

### Clinicopathological analysis of CHOL and COVID-19-related genes

The survival rates of the CHOL patients were correlated to CHOL/COVID-19-associated genes using the “survival” package in R as previous described [23]. Then the univariate and multivariate Cox proportional hazards regression analyses were used to determine the prognostic value of CHOL/COVID-19-associated genes. Finally, the patients were classified as low- and high-risk groups based on the average risk score as previous described [27].

### Determination of VA-pharmacological target in CHOL/COVID-19

In an attempt to determine the targets of VA, we searched online databases and tools, including the Traditional Chinese Medicine Systems Pharmacology Database and Analysis Platform (TCMSP), Swiss Target Prediction, TargetNet, Batman, Drugbank, and HitPick. The genes were used to compare with CHOL/COVID-19-associated genes. The overlapped genes were corrected using Swiss-Prot and the UniProt database with human settings as previous described [28, 29].

### Gene ontology enrichment and gene networking analyses of VA against CHOL/COVID-19-associated genes

The identified VA/CHOL/COVID-19-associated genes were subjected to Gene Ontology (GO) enrichment analysis and Kyoto Encyclopedia of Genes and Genomes (KEGG) pathways using R language packages. GO terms with and *p* value < 0.05 were considered as statistically significant. The association between VA/CHOL/COVID-19-associated genes, GO terms, and pathways was visualized using Cytoscape software (3.7.1 version) as previous described [21, 30]. Then the identified genes were subjected to the STRING database (version 11.0) for gene networking analysis to determine the protein-protein interactions (PPI) network map as previous described [31, 32].

### Metabolic pathway analysis

MetaboAnalyst 4.0 was used to analyze the metabolic pathway using the treatment targets of VA against CHOL/COVID-19. Further, the anti-CHOL/COVID-19 metabolic pathways were obtained using parameter determination in the metabolic pathway (integrated) database [33].

## RESULTS

### Collection of CHOL/COVID-19-associated genes

**Figure 1:**
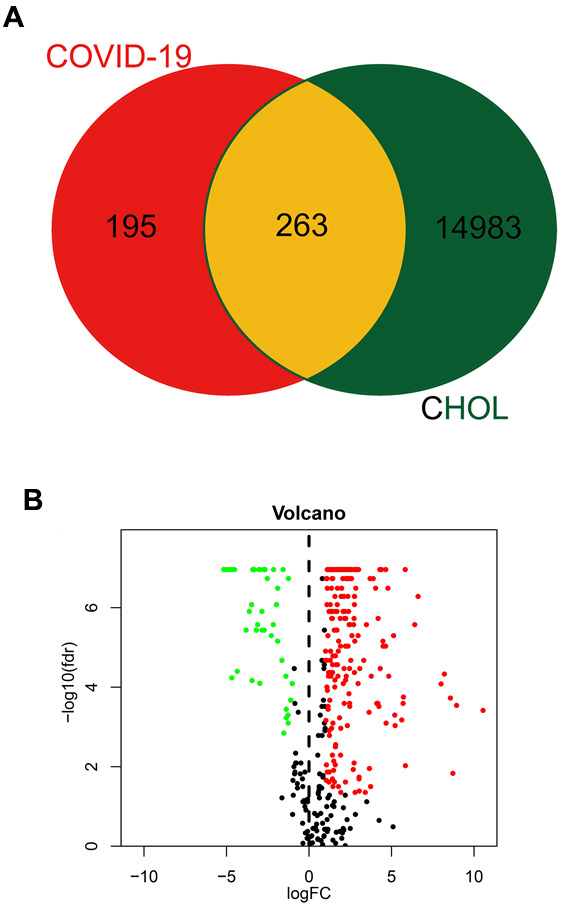
**Identification of CHOL/COVID-19-assocaited genes.** (**A**) Venn diagram depicted the number of intersecting genes in CHOL/COVID-19. (**B**) Volcano-plot showed the expression level of differential expressed genes (DEGs) found in CHOL. The genes with |log2 (fold change)| > 1 and -log10(FDR) > 1.3 were considered as DEGs.

Using the network pharmacology approach, we identified 458 genes associated with COVID-19 (Figure 1A). Meanwhile, 15,246 common differential expressed genes in CHOL patients were obtained from the TCGA database (Figure 1A). When we compared these two gene clusters, we found 263 overlapping genes between COVID-19 and CHOL patients (Figure 1A), in which, 221 genes were up-regulated and 42 genes were down-regulated in CHOL patients (Figure 1B).

### Clinical and medical analyses of CHOL/COVID-19-associated genes

**Figure 2:**
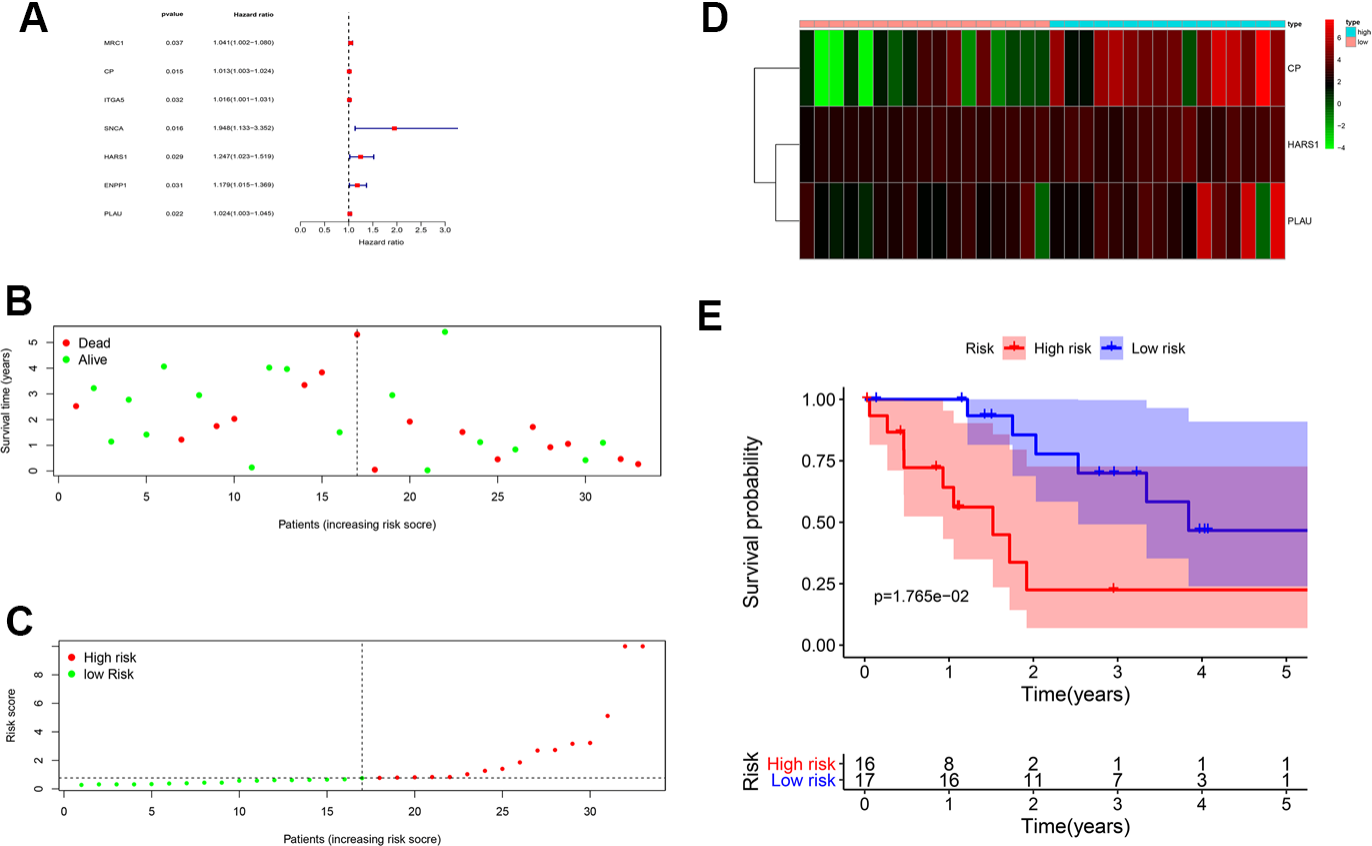
**Prognostic value of CHOL/COVID-19-associated genes.** (**A**) Univariate Cox analysis of 7 CHOL/COVID-19-associated genes, including *MRC1, CP, ITGA5, SNCA, HARS1, ENPP1,* and *PLAU*. (*p* < 0.05). Hazard ratio represented the correlation of the identified genes and CHOL. (**B**) Survival analysis indicated no difference in the overall survival between high- and low-risk groups of CHOL patients. (**C**) Analysis of patients’ risk score using Cox proportional hazards regression showed the increasing risk score in the CHOL patients with high risk. (**D**) Heatmap showed the overexpression of *CP, HARS1* and *PLAU* in the CHOL patients with high risk as compared to those with low risk. (**E**) The CHOL patients from high-risk group had a poor overall survival rate as compared to those from low-risk group.

To further reveal the clinical characteristics and the clinicopathological value of CHOL/COVID-19–associated genes, the 263 differential genes were subjected to univariate and multivariate Cox analyses. The univariate Cox analysis highlighted that 7 genes (including *MRC1, CP, ITGA5, SNCA, HARS1, ENPP1,* and *PLAU*) were significantly (*p* < 0.05) associated with CHOL/COVID-19 (Figure 2A and Table 1). Additionally, multivariate Cox analysis identified 3 target genes *CP, HARS1,* and *PLAU* (Table 2). The patients were divided into high- and low-risk groups based on the coefficient values of multivariate Cox proportional hazards regression analysis (Figure 2B and Table 2). In addition, we found a greater risk value in patients correlated with a higher risk score (Figure 2C) and it is related to the increased expression levels of CP, HARS1, and PLAU (Figure 2D). Furthermore, we conducted an independent single factor and multifactor prognostic analysis with the 3 genes. In the survival analysis, the data showed that the high- and low-risk groups related to these 3 genes had a significant impact on the overall survival (Figure 2E). Moreover, we also performed a univariate and multivariate independent prognostic analysis of the 3 genes. The difference in the independent prognostic analysis of the risk value was significant (p < 0.05), and the hazard ratio was greater than 1, indicating that as the risk value increases, the prognostic risk increases too. These results could be used as independent prognostic analysis factors for CHOL/COVID-19 (Table 3). The clinical correlation analysis of the 3 genes was carried out, and the results showed that there was no correlation between each gene and a single clinical factor (Table 4).

**Table 1:** Univariate Cox proportional hazards regression analysis of CHOL/SARS-CoV-2 gene.

**Symbol**	**HR**	**HR.95L**	**HR.95H**	**pvalue**
MRC1	1.0405	1.0023	1.0802	0.0375
CP	1.0132	1.0025	1.0241	0.0155
ITGA5	1.0163	1.0014	1.0313	0.0316
SNCA	1.9485	1.1326	3.3522	0.0160
HARS1	1.2468	1.0233	1.5191	0.0286
ENPP1	1.1789	1.0151	1.3690	0.0310
PLAU	1.0241	1.0034	1.0451	0.0222

**Table 2:** Multivariate Cox proportional hazards regression analysis.

**Symbol**	**coef**	**HR**	**95% CI**	**pvalue**
CP	0.0148	1.015	1.003-1.0271	0.014
HARS1	0.2251	1.2525	1.0056-1.56	0.0445
PLAU	0.024	1.0243	1.0028-1.0463	0.0265

**Table 3:** Univariate analysis and multivariate analysis of the correlation of three differentially expressed genes with overall survival (OS) among the patients.

**Parameter**	**Univariate analysis**				**Multivariate analysis**		
	**HR**	**95% CI**	**pvalue**		**HR**	**95% CI**	**pvalue**
gender	1.0997	0.3463-3.4925	0.8720		1.2914	0.2859-5.8327	0.7396
Stage(Stage I- Stage IV)	1.1708	0.7421-1.847	0.4979		2.4575	0.2699-22.3759	0.4250
T(T1-T4)	1.2231	0.6104-2.4509	0.5700		0.6070	0.0537-6.857	0.6866
M(M0-M1)	0.6067	0.0776-4.7458	0.6340		0.2070	0.0119-3.6046	0.2799
N(N0-N1)	1.5266	0.3161-7.3722	0.5985		0.4902	0.0107-22.5015	0.7150
riskScore	1.2421	1.0524-1.466	0.0104		1.2268	1.0226-1.4718	0.0278

**Table 4:** Clinical correlation analysis.

**Symbol**	**Gender (male vs female)**	**Stage (stage I and II vs stage III and IV)**	**T (T1 and 2 vs T3 and 4)**	**M (M0 vs M1)**	**N (N0 vs N1)**
CP	-0.463(0.649)	0.057(0.956)	-0.181(0.863)	1.53(0.151)	-0.417(0.699)
HARS1	0.446(0.659)	1.181(0.265)	0.418(0.684)	0.843(0.453)	2.063(0.092)
PLAU	0.295(0.770)	-0.91(0.401)	-0.924(0.405)	-0.859(0.479)	-1.023(0.379)
Risk Score	-1.425(0.156)	-1.48(0.141)	-2.172(0.031)	-1.507(0.137)	-1.507(0.134)

### Harvesting VA targets and intersection with COVID-19 and CHOL

**Figure 3:**
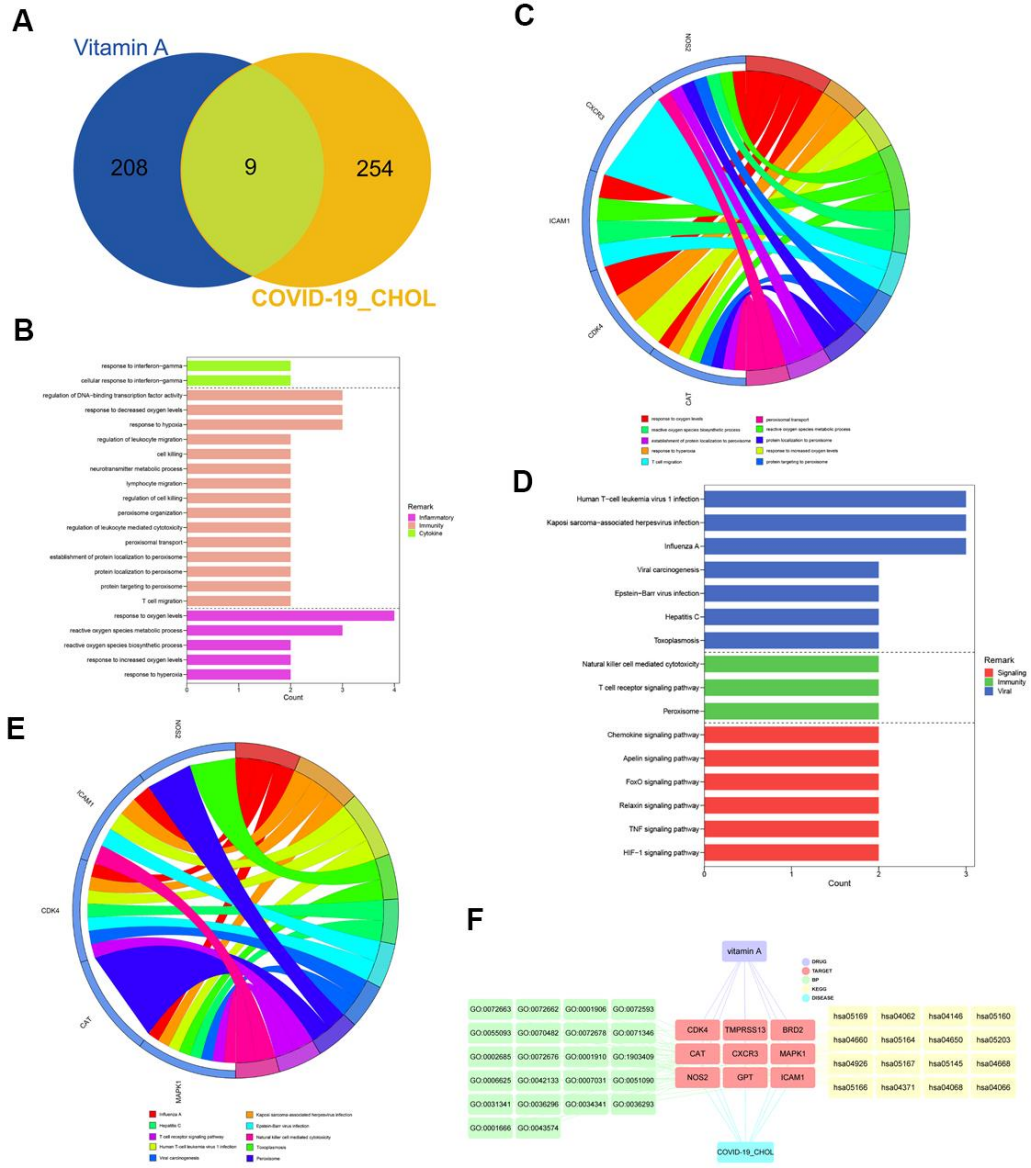
**Identification and functional characterization of CHOL/COVID-19/Vitamin A-associated genes.** (**A**) Venn diagram showed the number of intersecting genes of vitamin A and CHOL/COVID-19. (**B**) Gene ontology enrichment analysis highlighted the biological processes affected by the VA/CHOL/COVID-19-associated genes. (**C**) The bubble diagram showed the involvement of genes in different biological processes. (**D**) Kyoto Encyclopedia of Genes and Genomes (KEGG) pathway analysis demonstrated the alteration of cell signaling pathways by the VA/CHOL/COVID-19-associated genes. (**E**) The bubble diagram showed the involvement of genes in different cell signaling pathways. (**F**) Interaction network showed core biotargets, pharmacological functions, and signaling pathways of VA against CHOL/COVID-19.VA.

217 VA-related targets were obtained from the UniProt database. When we compared the CHOL/COVID-19-associated genes with VA-targeted genes, we identified 9 overlapping genes (Figure 3A and Supplementary Table 1). These 9 intersection genes were submitted to GO and KEGG enrichment analyses, the results showed that VA affected several biological processes related to oxygen level such as response to hypoxia, response to hyperoxia, and metabolic/biosynthetic process of reactive oxygen species. Also, our results highlighted T cell migration, peroxisomal protein targeting, protein localization to peroxisome, establishment of protein localization to peroxisome, peroxisomal transport, regulation of leukocyte-mediated cytotoxicity, peroxisome organization, regulation of DNA-binding transcription factor activity, regulation of cell killing, lymphocyte migration, neurotransmitter metabolic process, cell killing, regulation of leukocyte migration, and cellular response to interferon-gamma (Figure 3B, 3C and Supplementary Table 2). In the KEGG pathway analysis, 35 pathways related to Influenza A, Kaposi sarcoma-associated herpesvirus infection, human T-cell leukemia virus 1 infection, toxoplasmosis, hepatitis C, Epstein-Barr virus infection, viral carcinogenesis, peroxisome, T cell receptor signaling pathway, natural killer cell-mediated cytotoxicity, hypoxia-inducible factor-1 signaling pathway, tumor necrosis factor (TNF) signaling pathway, relaxin signaling pathway, FOXO signaling pathway, apelin signaling pathway, and chemokine signaling pathway were identified (Figure 3D, 3E and Supplementary Table 3). As a result, the network visualization of VA/CHOL/COVID-19 mediated biological processes and KEGG pathways was plotted using Cytoscape 3.7.1, as shown in Figure 3F.

### Identifying core targets of VA against CHOL and COVID-19

**Figure 4:**
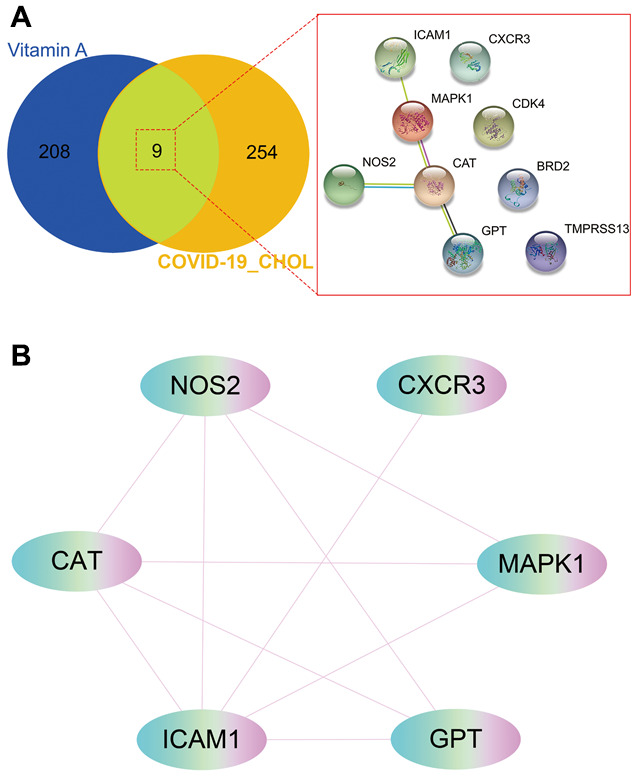
**Gene network analysis of vitamin A against CHOL/COVID-19.** (**A**) STRING analysis indicated protein-protein interaction mediated by 9 intersecting genes of VA against CHOL/COVID-19. (**B**) Cytoscape analysis further showed the involvement of 6 core candidates including *CAT*, *NOS2*, *CXCR3*, *MAPK1*, GPT, and *ICAM1* in protein interaction network related to action of VA against CHOL/COVID-19.

The 9 intersection targets of VA against CHOL/COVID-19 were subjected to STRING analysis to understand the protein-protein interaction (Figure 4A). Furthermore, six core gene targets including *CAT*, *NOS2*, *CXCR3*, *MAPK1*, GPT, and *ICAM1* of VA against CHOL/COVID-19 were identified using Cytoscape tool (Figure 4B).

### Findings of metabolic pathways in intersection targets

**Figure 5:**
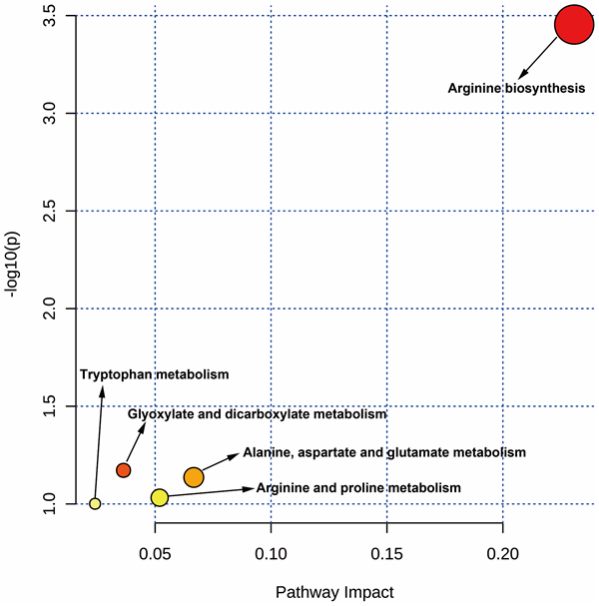
MetaboAnalyst analysis showed the targeted metabolic pathways by VA against CHOL/COVID-19.

Using the MetaboAnalyst tool, it was observed that the metabolic pathways of VA against CHOL/COVID-19 were involved in arginine biosynthesis; glyoxylate and dicarboxylate metabolism; alanine, aspartate, and glutamate metabolism; arginine and proline metabolism, and tryptophan metabolism (Figure 5).

## DISCUSSION

SARS-CoV-2, a new fatal virus, is of great concern in human health domain since its outbreak. This virus has spread rapidly without any national boundary, causing several deaths and economic losses [34, 35]. Although some vaccines against SARS-CoV-2 are available, their efficiencies still varied. Thus, the risk of infection and death from COVID-19 is still a global health problem [9]. In recent decades, chronic diseases, including cancers, are increasing yearly worldwide [36]. Cancer patients are immunosuppressed and have a heterogeneous immunity [37], leading to an increased risk for other infections. Hospital-acquired COVID-19 is increasing due to the high risk and exposure, especially in less-developed countries/areas [38]. In the 2020 cancer statistics, liver cancer is the main leading cause of cancer-related deaths in the United States and China [39]. As reported, the COVID-19 prevalence and deaths in the United States are increasing [40]. Accordingly, the patients with CHOL are at a high risk of being infected with SARS-CoV-2 due to the absence of an effective treatment. In addition, the therapeutic efficacy of existing pharmacotherapy will be further reduced in patients with simultaneous CHOL and COVID-19, resulting in an increase in the death rate.

It has been suggested that VA has an anti-proliferative property against liver cancer cells [41]. Moreover, given the potential anti-infective action of VA, it has been hypothesized that VA is likely effective in CHOL patients infected with SARS-CoV-2. In this bioinformatics analysis, all putative and core genes, and 263 mapped genes of CHOL/COVID-19 were identified. The DGE analysis showed 221 up-regulated and 42 down-regulated genes in patients with CHOL and/or COVID-19, suggesting the biomarkers for clinical characterization of CHOL patients with COVID-19. As per the independent prognostic and survival analyses, few of the important differentially expressed genes, including *MRC1, CP, ITGA5, SNCA, HARS1, ENPP1,* and *PLAU* may function as potent biomarkers for screening and characterizing different stages of CHOL patients with COVID-19. For instance, mannose receptor C-type 1 (MRC1) is a C-type lectin present on the surface of macrophages [42]. Genome-wide association studies demonstrated the importance of MRC1 in both of innate and adaptive immunity [43]. In addition, MRC1 coordinated with the activation of STAT6 for the differentiation of monocytes into monocyte-derived macrophages [44]. Integrin Subunit Alpha 5 (ITGA5), a family member of integrin alpha chain, plays role in cell-surface mediated signaling [45]. It has been reported that ITGA5 was a new candidate for SARS-CoV-2 cell binding and entry [46]. A transcriptome profile analysis also showed the overexpression of ITGA5 in lung samples from COVID-19 patients [47]. Synuclein Alpha (SNCA), a synuclein protein, is abundant in presynaptic terminals [48]. It has been reported the protective role of SNCA against SARS-CoV-2 infections in patients with Parkinson's disease [49].

Using the network pharmacology approach, we further identified 9 intersection genes of VA/CHOL/COVID-19. Moreover, the expression of *ICAM1, NOS2,* and *CAT* was increased in CHOL/COVID-19, while the survival rate was low, although these genes were only marginally increased. But it has been reported that these genes were contributed to the tumorigenicity of liver cancer. For instance, catalase (CAT) a key antioxidant enzyme defense against oxidative stress which played important role in the development of many cancer types [50]. Intercellular Adhesion Molecule 1 (ICAM1) is an oncogene of liver cancer. A microarray study of liver cancer patients showed that ICAM1 axis is necessary for tumor immune evasion and the tumorigenesis of liver cancer [51]. RNA-seq analysis revealed downregulation of the MAPK/ERK pathway through the downstream effectors ICAM1, leading to suppress tumor growth and increase chemosensitivity of liver cancer toward chemotherapy [52]. ICAM1 inhibition could suppresses tumor growth and metastasis [53]. A prospective cohort study of 282 patients with liver disease also demonstrated the association of soluble serum ICAM1 and liver cancer development [54]. Another clinicopathological study of 236 liver cancer patients suggested that ICAM promoted liver cancer metastasis and high serum ICAM1 level had shorter DFS and OS after resection in patients with liver cancer [55]. Nitric oxide synthase 2 (NOS2) was mainly expressed in liver and was found to provide crucial signals for angiogenesis in the tumor microenvironment [56]. It has been reported that the presence of NOS2 in mitochondria of liver cancer cells was associated with more aggressive phenotypes of cancer cells [57]. Because NOS2 was closely correlated with chronic inflammation and hepatocarcinogenesis in liver cancer [58]. These results suggested that the identified intersection genes may serve as pharmacological targets of VA against CHOL and COVID-19.

Lastly, the metabolic analysis highlighted some possible alterations of metabolic pathways in CHOL/COVID-19 patients. For instance, arginine biosynthesis and metabolism were found to be deregulated in our analysis. Arginine is an α-amino acid that is used for the protein synthesis. It was reported that accumulation of arginine metabolites by liver cancer cells is an important feature of non-alcoholic steatohepatitis-associated hepatocarcinogenesis [59]. A metabolomic study in mouse also demonstrated the arginine dynamics during hepatocellular carcinoma progression [60], suggesting the importance of arginine in the development of liver cancer. Other than arginine, tryptophan metabolism was also found to be induced in our analysis. It was concordant to the published literature that tryptophan was dramatically increased in liver cancer patients compared with healthy subjects [61]. And tryptophan metabolism was reported to be associated with metastasis and invasion of liver cancer [62].

The results of GO and KEGG enrichment analysis showed that the effects of VA on anti-CHOL and anti-COVID-19 were mainly through the regulation of immune responses such as anti-viral and anti-inflammatory actions, immunoregulation, influenza A, human T-cell leukemia virus 1 infection, viral carcinogenesis, T cell receptor signaling, natural killer cell-mediated cytotoxicity, TNF signaling, and chemokine signaling. Additionally, the anti-CHOL/COVID-19 effect of VA was controlled by 3 core genes, including *ICAM1, NOS2,* and *CAT,* suggesting the possible therapeutic and immunotherapeutic targets for treating COVID-19 or CHOL/ COVID-19.

## CONCLUSIONS

This study uncovers potential targets/pathways of VA treatment in CHOL/COVID-19, including the anti-viral and anti-inflammatory pathways, and immunopotentiation. This report, for the first time, suggested that VA is an alternative option for treating CHOL/COVID-19. However, further clinical studies are necessary to secure the clinical use of VA against CHOL/COVID-19.

## Supplementary Material

Supplementary Tables
