# Supplementary material for: Clinical characterization and therapeutic targets of vitamin A in patients with hepatocholangiocarcinoma and coronavirus disease
Source: Aging (Albany NY). 2021 Jun 27;13(12):15785–800. doi: 10.18632/aging.203220 (PMC8266307; doi:10.18632/aging.203220)
Supplement: Supplementary Tables [file aging-13-203220-s001.pdf]

SUPPLEMENTARY TABLES

Supplementary Table 1. List of CHOL/COVID-19/VA-associated genes.

Query	Entrez	Symbol	Name	Comment
BRD2	6046	BRD2	bromodomain containing 2	1
NOS2	4843	NOS2	nitric oxide synthase 2	1
GPT	2875	GPT	glutamic--pyruvic transaminase	1
MAPK1	5594	MAPK1	mitogen-activated protein kinase 1	1
CXCR3	2833	CXCR3	C-X-C motif chemokine receptor 3	1
ICAM1	3383	ICAM1	intercellular adhesion molecule 1	1
CDK4	1019	CDK4	cyclin dependent kinase 4	1
CAT	847	CAT	catalase	1
TMPRSS13	84000	TMPRSS13	transmembrane serine protease 13	1

Supplementary Table 2. Alteration of bioloigcal processes.

ONTOLOGY	ID	Description	GeneRatio	BgRatio	pvalue	p.adjust	qvalue	geneID	Count	Remark
BP	GO:0070482	response to oxygen levels	4/9	394/18670	2.26E-05	0.001975	0.001018	NOS2/ICAM1/ CDK4/CAT	4	Inflammatory
BP	GO:0055093	response to hyperoxia	2/9	21/18670	4.32E-05	0.003348	0.001727	CDK4/CAT	2	Inflammatory
BP	GO:0036296	response to increased oxygen levels	2/9	28/18670	7.76E-05	0.004923	0.002539	CDK4/CAT	2	Inflammatory
BP	GO:0072593	reactive oxygen species metabolic process	3/9	284/18670	0.000273	0.013629	0.007029	NOS2/ICAM1/ CAT	3	Inflammatory
BP	GO:1903409	reactive oxygen species biosynthetic process	2/9	122/18670	0.00148	0.024627	0.012702	NOS2/ICAM1	2	Inflammatory
BP	GO:0072678	T cell migration	2/9	65/18670	0.000423	0.01512	0.007798	CXCR3/ICAM1	2	Immunity
BP	GO:0006625	protein targeting to peroxisome	2/9	68/18670	0.000463	0.01512	0.007798	NOS2/CAT	2	Immunity
BP	GO:0072662	protein localization to peroxisome	2/9	68/18670	0.000463	0.01512	0.007798	NOS2/CAT	2	Immunity
BP	GO:0072663	establishment of protein localization to peroxisome	2/9	68/18670	0.000463	0.01512	0.007798	NOS2/CAT	2	Immunity
BP	GO:0043574	peroxisomal transport	2/9	69/18670	0.000477	0.01512	0.007798	NOS2/CAT	2	Immunity
BP	GO:0001666	response to hypoxia	3/9	359/18670	0.000543	0.01634	0.008427	NOS2/ICAM1/ CAT	3	Immunity
BP	GO:0036293	response to decreased oxygen levels	3/9	370/18670	0.000593	0.01634	0.008427	NOS2/ICAM1/ CAT	3	Immunity
BP	GO:0001910	regulation of leukocyte mediated cytotoxicity	2/9	78/18670	0.000609	0.01634	0.008427	NOS2/ICAM1	2	Immunity
BP	GO:0007031	peroxisome organization	2/9	81/18670	0.000656	0.016763	0.008646	NOS2/CAT	2	Immunity
BP	GO:0051090	regulation of DNA-binding transcription factor activity	3/9	432/18670	0.000931	0.019116	0.009859	MAPK1/ICAM1/ CAT	3	Immunity
BP	GO:0031341	regulation of cell killing	2/9	98/18670	0.000959	0.019116	0.009859	NOS2/ICAM1	2	Immunity
BP	GO:0072676	lymphocyte migration	2/9	111/18670	0.001227	0.021964	0.011328	CXCR3/ICAM1	2	Immunity
BP	GO:0042133	neurotransmitter metabolic process	2/9	153/18670	0.002313	0.032948	0.016993	NOS2/ICAM1	2	Immunity
BP	GO:0001906	cell killing	2/9	168/18670	0.00278	0.038807	0.020015	NOS2/ICAM1	2	Immunity
BP	GO:0002685	regulation of leukocyte migration	2/9	196/18670	0.003761	0.046872	0.024175	CXCR3/ICAM1	2	Immunity
BP	GO:0071346	cellular response to interferon-gamma	2/9	180/18670	0.003183	0.043191	0.022276	NOS2/ICAM1	2	Cytokine
BP	GO:0034341	response to interferon-gamma	2/9	199/18670	0.003874	0.047438	0.024467	NOS2/ICAM1	2	Cytokine

Supplementary Table 3. Alteration of KEGG pathways.

ID	Description	GeneRatio	BgRatio	pvalue	p.adjust	qvalue	geneID	Count	Remark
hsa05164	Influenza A	3/7	171/8047	0.00031	0.014567	0.009679	MAPK1/ICAM1/CDK4	3	Viral
hsa05167	Kaposi sarcoma-associated herpesvirus infection	3/7	189/8047	0.000416	0.014676	0.009751	MAPK1/ICAM1/CDK4	3	Viral
hsa05166	Human T-cell leukemia virus 1 infection	3/7	219/8047	0.000642	0.015083	0.010021	MAPK1/ICAM1/CDK4	3	Viral
hsa05145	Toxoplasmosis	2/7	112/8047	0.003852	0.025864	0.017185	NOS2/MAPK1	2	Viral
hsa05160	Hepatitis C	2/7	156/8047	0.007356	0.034574	0.022972	MAPK1/CDK4	2	Viral
hsa05169	Epstein-Barr virus infection	2/7	202/8047	0.012117	0.049748	0.033054	ICAM1/CDK4	2	Viral
hsa05203	Viral carcinogenesis	2/7	204/8047	0.012349	0.049748	0.033054	MAPK1/CDK4	2	Viral
hsa04146	Peroxisome	2/7	83/8047	0.002134	0.021497	0.014283	NOS2/CAT	2	Immunity
hsa04660	T cell receptor signaling pathway	2/7	104/8047	0.00333	0.025864	0.017185	MAPK1/CDK4	2	Immunity
hsa04650	Natural killer cell mediated cytotoxicity	2/7	131/8047	0.005235	0.029527	0.019619	MAPK1/ICAM1	2	Immunity
hsa04066	HIF-1 signaling pathway	2/7	109/8047	0.003652	0.025864	0.017185	NOS2/MAPK1	2	Signaling
hsa04668	TNF signaling pathway	2/7	112/8047	0.003852	0.025864	0.017185	MAPK1/ICAM1	2	Signaling
hsa04926	Relaxin signaling pathway	2/7	129/8047	0.00508	0.029527	0.019619	NOS2/MAPK1	2	Signaling
hsa04068	FoxO signaling pathway	2/7	131/8047	0.005235	0.029527	0.019619	MAPK1/CAT	2	Signaling
hsa04371	Apelin signaling pathway	2/7	137/8047	0.005713	0.030985	0.020587	NOS2/MAPK1	2	Signaling
hsa04062	Chemokine signaling pathway	2/7	189/8047	0.010662	0.045556	0.030269	MAPK1/CXCR3	2	Signaling
